# Buffalo Medical and Surgical Association

**Published:** 1890-04

**Authors:** W. H. Bergtold

**Affiliations:** Secretary


### Society Proceedings.

### BUFFALO MEDICAL AND SURGICAL ASSOCIATION.

Reported by W. H. BERGTOLD, M D., Secretary.

The postponed monthly meeting was held on Tuseday evening, Feb. 11th, at nine o'clock, in the Library of the Polytechnic Institute, No. 9 West Mohawk street.

The president, Dr. A. A. HUBBELL, in the chair.

Dr. W. S. RENNER read a paper entitled, Adenoid Vegetations of the Naso-Pharynx. See page 523.

Dr. M. D. MANN read next, A Report of 160 Abdominal Sections, with Special Reference to the Prevention of Unpleasant Sequelæ. See page 535.

### DISCUSSION.

Dr. HERMAN MYNTER, in relation to Dr. Mann's paper, said that the question of the occurrence of ventral herniæ after hernia operations had perplexed him not a little. Laterally he has had four cases where, after operation on a hernia, exsecting the sac, sewing with catgut and silver, the patients returned later with ventral hernias. He thinks that silver wire does not do well here, just as in inguinal hernia. He now discards the use of McEwan's operation, and uses, instead, McBurney's method, that of removing the sac entirely, and allowing the wound to fill by granulation. He asked how many of Dr. Mann's cases had developed ventral hernia? What does he do to prevent this complication, and how does he treat them?

Dr. H. E. Hayd thought that the light death-rate in the essayist's last fifty cases, as compared with it in the first fifty, could be, perhaps, taken as an index of what experience will do for an operator's results. He asked the writer whether he would operate so often, or on the same cases to-day, as he did several years ago? Dr. Hayd spoke of Apostoli's method of treating fibroids, etc., and maintained that it would, in many cases, supplant laparatomy.

Dr. H. D. Ingraham asked Dr. Mann if he did not believe that one could use silk just as well as gut, if it were properly prepared. He also asked how long Dr. Mann allowed the sponges to remain in the abdomen, when used to check oozing.

Dr. M. Hartwig believed that drainage is of immense value. It is chosen through the abdominal walls, in laparatomy, because when conducted by way of the vagina it almost surely involved septicemia. Drainage *per vaginam* gives us the benefit of gravity, and, furthermore, by only opening into the *cul-de-sac*, we, in a manner, do not meddle with the major part of the peritoneal sac; this way, if properly conducted, is exceedingly useful, and ought to be perfected, if possible. He believes that ventral hernia can be avoided by careful apposition, and approximation of the abdominal walls and their different layers. Silk has the advantage that it can be made aseptic so quickly and easily.

Dr. Lucien Howe said that he one time used silk when advancing an ocular muscle, and it had demonstrated very completely how long silk will remain without undergoing change. In this case he was able to see and feel the silk suture for over six months. He thinks that the methods employed by Dr. Renner are very satisfactory, and have been of much benefit in several cases which he had referred to him.

Dr. M. D. Mann, in closing the discussion, and in reply, first to Dr. Mynter, said that he could at the moment recall four cases which presented afterward with ventral hernias. He knew of no case where the fascia had been stitched that this occurred. He closes the abdominal incision in the following way: First, the required number of silver wire sutures are inserted through the entire thickness of the wall, twisted and laid aside; then the various layers, as peritoneum, etc., are carefully adjusted, one edge to its fellow on the opposite side by continuous gut stitches, and finally the silver sutures are tightened firmly so as to relieve all tension from the gut sutures. In reply to Dr. Hayd, he said that on some of the cases of fibroids he would not now to-day operate, because electrolysis gives such good results, but in the cases of tube and ovary disease, any amount of electricity would not produce a normal condition, and that he would still operate in such cases. Dr. Ingraham was correct in saying that silk, if properly prepared, would cause no suppuration, but silk, if it happens to become situated at the bottom of a sinus, would never disappear, and it would cause such an opening to remain patulous; whereas catgut, if similarly situated, would, at the least, rot out, and not act as the continuing cause of the sinus. He leaves sponges, for the control of oozing, ten to fifteen minutes, and, if necessary, longer, though this has never been necessary. Personally, he is not satisfied with vaginal drainage, and believes that Sims gave it up after a thorough trial.

The President appointed Drs. Hayd, Samo and Coakley a committee to draft resolutions on the death of Dr. P. H. Strong.

The essayists for the next meeting were announced as Dr. H. E. Hayd, subject, Electricity in Gynecological Practice, and Dr. Roswell Park, subject, Surgery of the Alimentary Canal, with Demonstrations.

